# Reply to Spavieri, J.H.P. et al. Comment on “Zieliński, G.; Gawda, P. Analysis of the Use of Sample Size and Effect Size Calculations in a Temporomandibular Disorders Randomised Controlled Trial—Short Narrative Review. *J. Pers. Med.* 2024, *14*, 655”

**DOI:** 10.3390/jpm15030086

**Published:** 2025-02-26

**Authors:** Grzegorz Zieliński, Piotr Gawda

**Affiliations:** Department of Sports Medicine, Medical University of Lublin, 20-093 Lublin, Poland; piotr.gawda@umlub.pl

We have received a request to respond to a comment on our article published in the *Journal of Personalized Medicine* (2024; 14: 655) [1].

To begin, we would like to thank the authors of the comment for their observations and for initiating a scholarly discussion [2].

At the request of the commenters, we re-evaluated Table 2 [1] in light of a scoring error in the work of Aguiar et al. (2024) [3]. Upon re-analysis of the study and review of the table, we acknowledge that there was an error in our analysis. In this response, we wish to apologize to all the authors of the article (Aroldo d.S. Aguiar, G. Lorimer Moseley, Cesar Bataglion, Beatriz Azevedo, Thaís C. Chaves) for our oversight [3]. We would also like to note that a correction associated with this error will be submitted to the *Journal of Personalized Medicine*.

Regarding the remaining part of the comment, we thank the authors for raising and further considering the use of effect size calculations. To address this, I would like to outline a few dates and historical events.

In 1996, the Task Force on Statistical Inference (TFSI) was convened by The Board of Scientific Affairs (BSA) of the American Psychological Association (APA) [4]. In 1999, Wilkinson and TFSI issued recommendations titled “Statistical methods in psychology journals: Guidelines and explanations”, published in *American Psychologist*, 54(8), 594–604 [5]. At the time, researchers were encouraged to report effect sizes [5], as effect size calculations provide additional insights about the test outcomes that traditional null hypothesis significance testing cannot offer. For instance, they give an estimate of the magnitude of difference or the strength of association [6].

In 2012, an editorial was published by Gail M. Sullivan, MD, MPH, Editor-in-Chief of the *Journal of Graduate Medical Education*, and Richard Feinn, PhD, Assistant Professor in the Department of Psychiatry at the University of Connecticut Health Center. This article, titled “Using Effect Size—or Why the *p* Value Is Not Enough” [7], has been widely acknowledged within the scientific community, cited 6190 times as of the date of our response to the comment on our article [7]. The editors explained why effect size reporting is essential, using the case of aspirin use for myocardial infarction prevention [8]. Sullivan and Feinn concluded the following:

“*Effect size helps readers understand the magnitude of differences found, whereas statistical significance examines whether the findings are likely to be due to chance. Both are essential for readers to understand the full impact of your work. Report both in the Abstract and Results sections.*”

[7]

In 2013, Lakens published a work titled “Calculating and reporting effect sizes to facilitate cumulative science: a practical primer for *t*-tests and ANOVAs” [9]. This article has since received even greater response from the scientific community, having been cited 9415 times as of the time of our response to the comment [9]. Lakens began his work with the following statement:

“*Effect sizes are the most important outcome of empirical studies.*”

[9]

Examples of recommendations on the use of effect size calculations are abundant, with new guidelines continuously being issued for research [6,10,11], as noted in our own study [1].

The *p*-value of 0.05 means that, assuming the null hypothesis is true, there is a 5% chance of obtaining results as extreme as, or more extreme than, the observed results. This is a commonly accepted threshold for significance in scientific research. However, the *p*-value alone does not tell us whether the result has real clinical relevance. There are factors that can influence the achievement of statistical significance. For example, we might be lucky in a study and recruit participants with characteristics that increase the likelihood of observing an effect. Another factor is sample size—in very large groups of participants, it is easier to detect statistical significance, even when the actual effect is very small.

In both of these cases, statistical significance (i.e., *p* < 0.05) may be achieved, but this does not automatically mean that the result has clinical value. Therefore, it is crucial to consider the effect size, which indicates how large the differences between groups are or how strong the relationship between variables is. Effect size helps determine whether the observed differences are not only statistically significant but also clinically meaningful.

From both clinical and methodological perspectives, effect size plays a key role. It is essential for advanced analyses such as meta-analyses or meta-regressions, as, without some form of effect size calculation, reliable conclusions about combined study results cannot be drawn [12,13,14,15]. The importance of effect size can be illustrated with examples. Examples were included in our prior publication [1], but let us revisit a few in this paper, particularly the most commonly cited one by the previously mentioned editors, Sullivan and Feinn, regarding the work of Bartolucci et al. [8]. Once again, I will directly quote a passage from Sullivan and Feinn [7]:

“*In more than 22 000 subjects over an average of 5 years, aspirin was associated with a reduction in MI (although not in overall cardiovascular mortality) that was highly statistically significant: p < 0.00001. (…) However, the effect size was very small: a risk difference of 0.77% with r^2^ = 0.001—an extremely small effect size.*”

[7]

Of course, this pertained to cardiology, but studies on TMDs (temporomandibular disorders) are not isolated in terms of the importance of effect size. For example, the authors Hanna et al. emphasized the following in their conclusions:

“*When we used the standardized effect size estimate to determine whether TMD experience is clinically relevant to our participants’ OHRQoL (oral health-related quality of life) in an adjusted analysis, we found that it was of a small clinical relevance.*”

[16]

This highlights the critical role of effect size in drawing conclusions.

Even a small effect size is not disqualifying for results, but it should be noted, as it provides other researchers and clinicians with valuable information about the need for further analysis and studies. An example can be found in Márquez-Vera et al.’s study, which aimed to evaluate the effects of a specific manual therapy technique (the mandibular muscle energy technique) in adults with TMDs: despite achieving a statistically significant threshold (*p* < 0.001), the results demonstrated a small effect size [17]. Acknowledging this, the authors explicitly noted the following:

“*Therefore, these results should be interpreted with caution, especially considering that a weak effect size was demonstrated in this context.*”

[17]

This enables other researchers to conduct further analyses and test the techniques studied.

Using effect size can also be essential for evaluating results that are not statistically significant. For instance, in the study by Packer et al., the authors aimed to assess the impact of upper chest manipulation on vertical mouth opening (VMO) and electromyographic activity of masticatory muscles in women with TMDs [18]. The following is a quote from the authors’ conclusions:

“*In the present study, no significant differences were found regarding VMO between the experimental and placebo groups or among the different evaluations times in each group. Moreover, Cohen d test revealed no clinical effect of the technique.*”

[18]

Statistics, as international guidelines, apply to all studies. Let me emphasize once again that this is not about numbers but about drawing conclusions based on statistical results (sample size, effect size, *p*-values) for the benefit of patients. The lack of all variables limits comprehensive inference, which may result in incorrect guidelines for diagnostics and therapy. Taking into account the error we highlighted in the first paragraphs of this response, the percentage of use of these effect measures is still too low.

Continuing with my answer, in response to paragraphs 6–8 of the comment, given the above information, we wish to emphasize that effect size in scientific research is significant in and of itself, and interpreting its value is critical to assessing the practical relevance of findings. The effects observed in studies are neither empty nor devoid of meaning, even if they require context for a fuller understanding. Effect size should not be considered meaningless, as simply determining whether an effect is large or small enables a preliminary assessment of its importance, or lack thereof, for a given phenomenon [6,7,8,9,10].

The interpretation of effect size is not based solely on subjective judgment. Frameworks such as Cohen’s classification provide valuable context for effect size, serving as an essential reference point for researchers [19]. While it is true that the significance of an effect depends on context, the use of standardized benchmarks enables researchers to compare study results within similar fields. This means that preliminary interpretations based on these standards are neither random nor arbitrary; rather, they are tailored to the needs of the specific research area, thus objectifying data analysis [6,10,20,21].

The researcher’s role in interpreting results is undoubtedly important, but this does not imply that each researcher subjectively assesses the significance of the effect. A scientist’s professional experience may influence subtler interpretative nuances, yet statistical principles and analytical tools, including effect size, provide an objective foundation for assessment. Only by interpreting results in relation to other studies and accepted standards can one realize their full significance [22].

Moreover, effect size, beyond the context of *p*-values, is itself a critical step toward understanding research outcomes. The concept of a minimally important effect, or the smallest benefit deemed worthwhile by patients to pursue an intervention, is a valuable addition, as it allows for evaluating whether the effect observed in a study has real value for patients. This approach not only facilitates the assessment of outcomes but also bridges the gap between statistical analysis and practical clinical considerations [7,8].

We understand that not everyone may agree with our reasoning and the evidence presented in this response, as well as in our original article [1]. However, in the 21st century, with researchers having broad access to statistical software and online analysis resources (examples of which we cited in our article) [1], conducting effect size analyses should not present a significant burden to researchers. From a cost–benefit perspective, the decision of whether to include effect size calculations yields only benefits. Reporting effect size results can assist other researchers in understanding and comparing differences between studies, which can ultimately enhance patient care. This should serve as the primary goal of scientific endeavor—to better serve patients through faster diagnostics and improved therapies. In the context of our work, this applies to patients with temporomandibular disorders, a prevalent issue affecting approximately 34% of the global population, posing a significant health challenge [23].

In closing, we continue to encourage the reporting of sample sizes and effect sizes. Once again, we thank the authors of the comment for initiating this discussion. We apologize to the Aguiar et al. team for our error in evaluating their article.
